# Resting-state functional connectivity predicts motor cortex stimulation-dependent pain relief in fibromyalgia syndrome patients

**DOI:** 10.1038/s41598-022-21557-x

**Published:** 2022-10-12

**Authors:** Yuval Argaman, Yelena Granovsky, Elliot Sprecher, Alon Sinai, David Yarnitsky, Irit Weissman-Fogel

**Affiliations:** 1 Clinical Neurophysiology Lab, Bruce Rappaport Faculty of Medicine, Technion – Israel Institute of Technology, Haifa, Israel; 2 Department of Neurology, Rambam Health Care Campus, Haifa, Israel; 3 Department of Neurosurgery, Rambam Health Care Campus, Haifa, Israel; 4 Department of Physical Therapy, Faculty of Social Welfare and Health Sciences, University of Haifa, Haifa, Israel

**Keywords:** Neurophysiology, Predictive markers

## Abstract

MRI-based resting-state functional connectivity (rsFC) has been shown to predict response to pharmacological and non-pharmacological treatments for chronic pain, but not yet for motor cortex transcranial magnetic stimulation (M1-rTMS). Twenty-seven fibromyalgia syndrome (FMS) patients participated in this double-blind, crossover, and sham-controlled study. Ten daily treatments of 10 Hz M1-rTMS were given over 2 weeks. Before treatment series, patients underwent resting-state fMRI and clinical pain evaluation. Significant pain reduction occurred following active, but not sham, M1-rTMS. The following rsFC patterns predicted reductions in clinical pain intensity after the active treatment: weaker rsFC of the default-mode network with the middle frontal gyrus (r = 0.76, p < 0.001), the executive control network with the rostro-medial prefrontal cortex (r = 0.80, p < 0.001), the thalamus with the middle frontal gyrus (r = 0.82, p < 0.001), and the pregenual anterior cingulate cortex with the inferior parietal lobule (r = 0.79, p < 0.001); and stronger rsFC of the anterior insula with the angular gyrus (r =  − 0.81, p < 0.001). The above regions process the attentional and emotional aspects of pain intensity; serve as components of the resting-state networks; are modulated by rTMS; and are altered in FMS. Therefore, we suggest that in FMS, the weaker pre-existing interplay between pain-related brain regions and networks, the larger the pain relief resulting from M1-rTMS.

## Introduction

Pre-treatment resting-state functional connectivity (rsFC) of pain-related brain regions, can predict pharmacological and non-pharmacological responses to chronic pain treatments. For example, reduced pre-existing rsFC between the pregenual anterior cingulate cortex (pgACC) and posterior insula (pINS), periaqueductal Gray (PAG) with the mid-INS, and dorsolateral prefrontal cortex (dlPFC) with the inferior parietal lobule (IPL) predicted a more significant reduction in clinical pain following selective serotonin–norepinephrine reuptake inhibitor administration, which enhances antinociceptive processes^1^. Furthermore, decreased connectivity of the lateral thalamus with the pINS and the primary sensory and motor cortices (S1/M1, respectively) predicted greater clinical pain relief in fibromyalgia syndrome (FMS) following transcranial direct current stimulation (tDCS) of primary motor cortex M1^2^. Furthermore, such predictive connectivity patterns appear on larger connectivity scales, i.e., resting-state networks (RSNs). For example, greater connectivity of the INS and with the default-mode network (DMN) correlated with more potent pregabalin-dependent clinical pain reduction^3^. Overall, the above evidence suggests that a clinically relevant interplay among and between pain-processing areas, and high-order networks involvement, influence the efficacy of analgesic treatments.

Fibromyalgia syndrome (FMS) patients exhibit altered rsFC between pain-processing brain areas, as described above, and within and between RSNs^4^, compared to healthy individuals^5–7^. Evidence links altered DMN rsFC to FMS symptomatology^8^. For example, greater clinical pain intensity is associated with stronger DMN rsFC with the anterior insula (aINS), a salience network (SN) component^9,10^. Also, altered DMN-SN connectivity is related to symptom severity, bodily pain spread, and functional disability^11,12^. Furthermore, FMS symptomatology is associated with rsFC strength of pain-processing brain areas^13–16^ like the PAG, a prominent brainstem pain inhibitory area, with the prefrontal, anterior cingulate and aINS cortices modulating its activity^13,14^. Therefore, the aberrant rsFC between networks of brain areas underlying FMS symptomatology might serve as a treatment-response predictive chronic pain biomarker^17^.

Repetitive transcranial magnetic stimulation of the motor cortex (M1-rTMS) is a non-invasive therapy with proven long-term pain relief and daily function and quality-of-life improvements in FMS patients treated by the active, but not a sham, procedure^18,19^. The analgesic effects of TMS derive from: (i) activation of brain areas beyond the stimulated area^20^; and (ii) modulation of the connectivity of brain areas directly or indirectly involved in pain processing^21^, specifically cortical and sub-cortical brain areas in the ascending pain transmission and descending antinociceptive systems. FMS patients exhibit disruptions in these systems^22,23^, and the M1-rTMS-related analgesic function relies on them^24,25^. Furthermore, rTMS modifies domain-general networks e.g., DMN and the executive control network (ECN)^26^. However, it is not known whether pre-treatment rsFC can predict the M1-rTMS-dependent analgesic effects.

We investigated whether the strength of connectivity between networks of brain areas that are associated with FMS symptomatology and pain relief treatments could predict the M1-rTMS analgesic effects. We hypothesized that the following would predict greater analgesic potency: (i) weaker connectivity within the ascending pain transmission pathway based on the role of the lateral thalamus in the prediction of analgesic effects following M1 stimulation^2^ which suppresses pain stimuli by lateral thalamus inhibition^27,28^; (ii) weaker connectivity of antinociceptive brain areas, since rsFC between pain-modulatory brain areas predicted the analgesic effects associated with antinociceptive medication^1^; and (iii) stronger connectivity between DMN and SN components (based on Ref.^3^).

## Results

### Demographics

We screened 195 FMS patients. Of them, 145 individuals were excluded based upon interview for the following reasons: unwillingness or inability to participate—45; incompatibility with inclusion criteria—56; would not disclose reason or lost to initial contact—44. Fifty individuals were allocated to a randomized controlled trial. We excluded 23 patients after the allocation; 15 before treatment (withdraw consent-7, claustrophobia inside scanner-1, allocated to a different study-3, resting motor threshold exceeding safety limits-2, the pain stimuli in the psychophysical tests were too painful-2); 1 during the first session (developed tinnitus-1); 6 during washout (retired voluntary-4, discovered pregnancy-1, developed tinnitus-1); and 1 after study completion (lost imaging data). Overall, 27 female FMS patients completed, or had the available results from, all experimental and treatment phases. The participants were between ages 19–55 (median 38, mean ± SD 38.0 ± 10.6 years), with a median disease duration of 5.8 years (range 1–20 years). As we previously reported, we found no significant differences in age, disease duration, and frequency of pain prophylaxis between the included and excluded patients^21^.

### Active M1-rTMS is more effective than sham in reducing clinical pain

**Figure 1:**
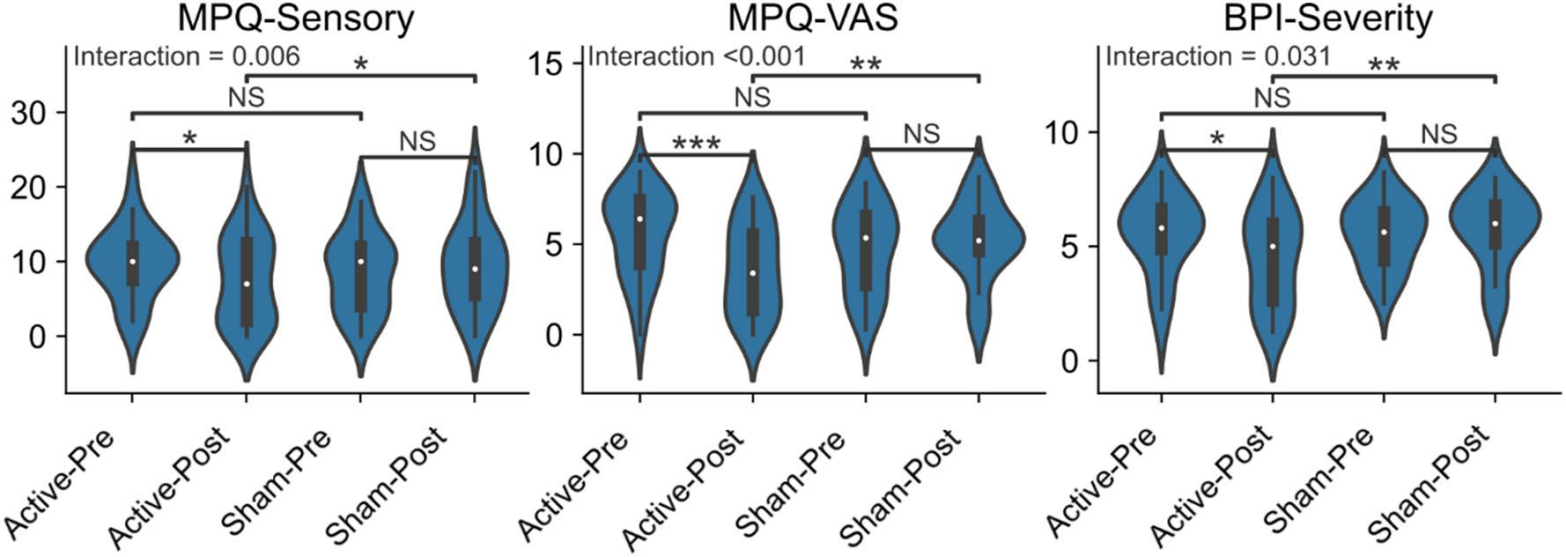
Active M1-rTMS is superior to sham in relieving clinical pain. Time × Treatment interaction in MPQ-Sensory was tested using Friedman’s ANOVA with Wilcoxon–Nemenyi–McDonald–Thompson post-hoc tests. All p-values are corrected for 4 preplanned contrasts. *BPI* Brief Pain Inventory, *MPQ* McGill Pain Questionnaire, *NS* not significant, *VAS* visual analog scale; *p < 0.05; **p < 0.01; ***p < 0.001.

We found significant time × treatment interactions in the following measures: MPQ-Sensory (p = 0.006), MPQ-VAS (p < 0.001), and BPI-Severity (p = 0.031). A post hoc test revealed that the active M1-rTMS was superior to sham in reducing clinical pain (Fig. 1).

### gICA

**Figure 2:**
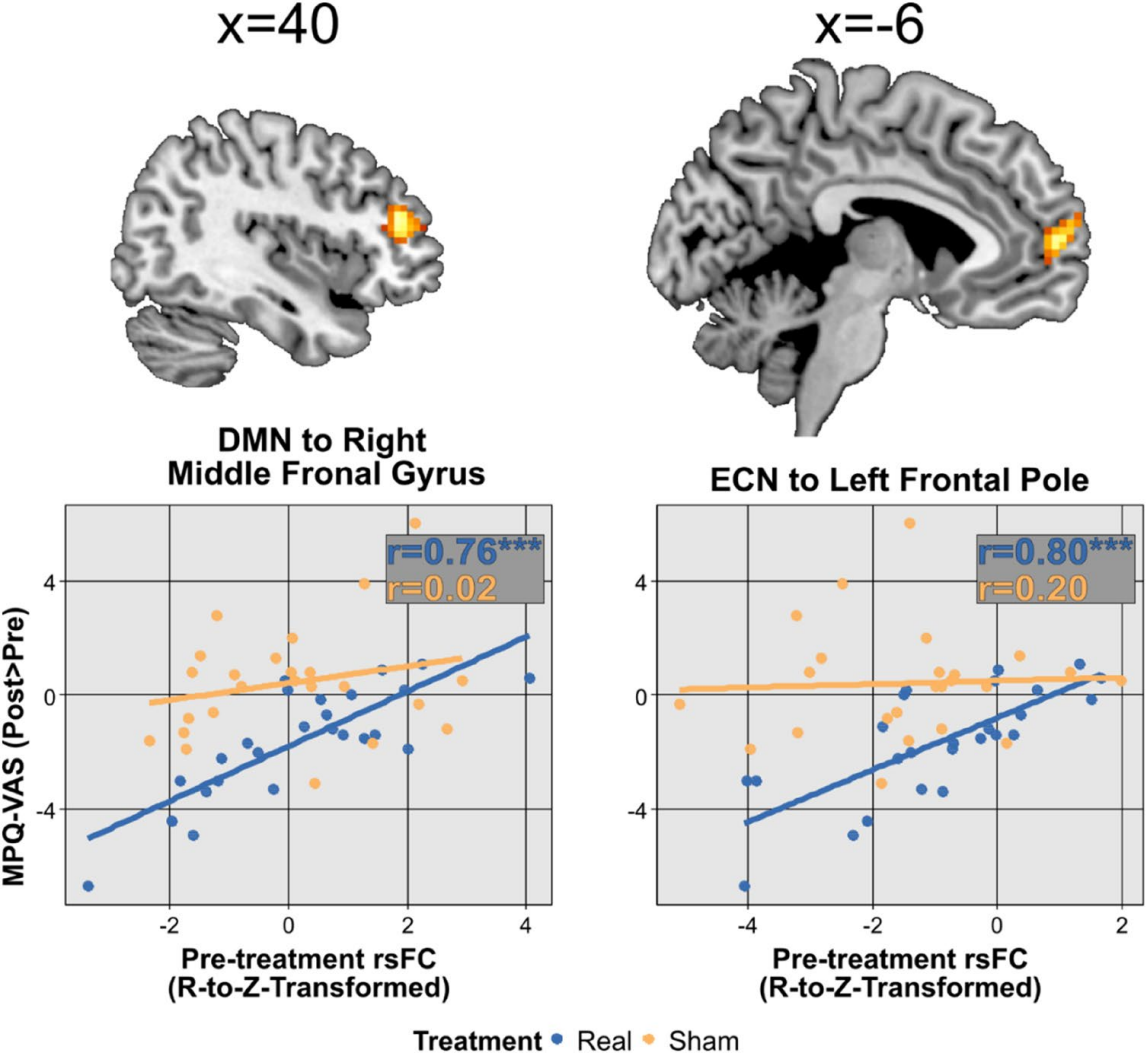
MPQ-VAS reduction following the real, but not sham M1-rTMS, was predicted by stronger resting-state FC of the DMN and ECN with the frontal pole. Coordinates in upper panels are in MNI space. *r* Pearson’s correlation coefficient; ***p < 0.001.

Information about the significant clusters appears in Table 1. Briefly, we found that changes in MPQ-VAS scores following real, but not sham, M1-rTMS, were predicted by: (i) weaker pre-treatment rsFC of the DMN with the right middle frontal gyrus (MFG) (Fig. 2, left panel); and (ii) of the ECN with the left frontal pole (Fig. 2, right panel). We found no other associations between network rsFC and changes in the other clinical parameters.

**Table 1:** Clusters exhibiting a significant association of baseline RSN connectivity with the change in MPQ-VAS scores following real M1-rTMS**.**

Network	Target (location, brain area)	FC (R-to-Z-transformed)	MNI (x, y, z)	Size (voxels)	pFDR
DMN	Right MFG, ventral dlPFC cortex	6.57	42, 44, 19	88	0.024
ECN	Left frontal pole, rmPFC	6.06	− 3, 59, 7	63	0.042

*DMN*, default-mode network, *dlPFC* dorso-lateral prefrontal cortex, *ECN* executive control network, *MFG* middle frontal gyrus, *vmPFC* rostro-medial prefrontal cortex.

### SCA

**Figure 3:**
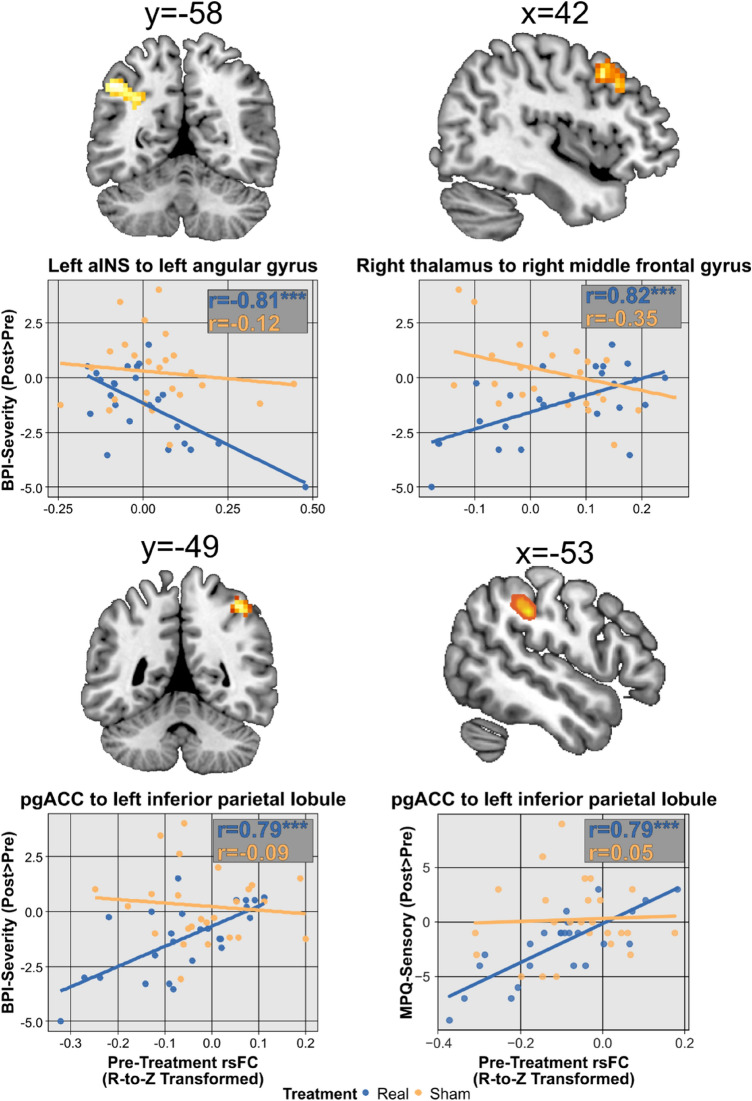
rsFC of ascending nociceptive and descending pain inhibition brain areas BPI-Severity predict reductions following real, but not sham, M1-rTMS. Coordinates in upper panels are in MNI space. *aINS* anterior insula r, Pearson’s correlation coefficient, *pgACC* pregenual anterior cingulate cortex. ***p < 0.001.

Information about pre-treatment SCA connectivity pairs relating to significant clinical changes following real M1-rTMS appears in Table 2. We found that reductions in BPI-Severity following real M1-rTMS, but not sham, were predicted by (i) stronger rsFC of the left aINS with the left angular gyrus (Fig. 3, upper left panel); (ii) weaker rsFC of the right thalamus with the right MFG (F = 20.20, p < 0.001) (Fig. 3, upper right panel); and (iii) weaker rsFC of the pgACC with the left inferior parietal lobule (Fig. 3, lower, left). Curiously, the last connectivity pattern also predicted more pronounced reductions in MPQ-Sensory ratings following the real, but not sham, M1-rTMS (Fig. 3, lower right panel).

**Table 2:** Clusters of SCA connectivity exhibiting significant associations of treatment response with real M1-rTMS.

Measure	Seed	Target	FC (R-to-Z-transformed)	MNI (x, y, z)	Size (voxels)	p-FDR
MPQ-Sensory	pgACC	Left inferior parietal lobule	5.63	− 51, − 34, 37	124	< 0.001
BPI-Severity	Left aINS	Left angular gyrus	− 5.17	− 45, − 58, 43	78	0.008
	pgACC	Left inferior parietal lobule	4.88	− 45, − 43, 49	60	0.044
	Right thalamus	Right middle frontal gyrus	6.49	45, 14, 46	95	0.001

FDR- and FWE-corrected p-values are also familywise-adjusted for the number of seeds in class.

*aINS* anterior insula, *pgACC* pregenual anterior cingulate cortex.

## Discussion

For the first time, we show that rsFC can predict M1-rTMS-dependent reduction in clinical pain. Overall, weaker rsFC predicted a more pronounced clinical effect following real M1-rTMS while it did not predict the sham treatment responses. The predictive connectivity patterns were identified in neural constructs previously implicated in FMS: the DMN, ECN, and pain brain areas such as the thalamus, pgACC, and aINS that potentially can be modulated by M1-rTMS^25^. Indeed, we previously reported that the rsFC of some of the predictive regions has changed following treatment^21^. Therefore, we provide evidence for the predictive value of neuroimaging in pain-related non-invasive brain stimulation, specifically in FMS.

An objective determinant or a set of such that could potentially point to appropriate treatment for chronic pain is the hallmark of precision pain medicine^29^. Recent reports suggest that the function of brain areas implicated in chronic pain might serve to determine the patient’s ability to benefit from analgesic treatments. Notably, these studies’ predictive regions appear in all parts of the central pain pathways (sensory transmitting, affective-motivational, and descending antinociceptive) and converge to nodes of the dynamic pain connectome^30^. For example, stronger rsFC of the dorsal ACC (affective-motivational) with the S2 (sensory) and M1 (motor) predicted neuropathic pain relief following administration of ∆9-tetrahydrocannabinol, the primary psychoactive compound in the cannabis plants^31^. However, weaker rsFC of the pgACC (descending antinociceptive and affective) with the pINS (sensory) and PAG (descending antinociceptive) predicted more pronounced clinical pain relief following duloxetine administration in FMS patients^1^. Next, stronger rsFC of the mPFC (descending antinociceptive, DMN) with the insula and basal ganglia (motor), and weaker rsFC of the mPFC with the angular gyri (AG) (association, DMN), predicted more significant clinical pain relief in chronic lower back pain patients^32^. Finally, in FMS patients, stronger rsFC of the ventrolateral thalamus (sensory) with the M1 and with the PAG, as well as of the S1 (sensory) with the aINS, were associated with better pain relief following tDCS^2^. The above evidence does not point to strict heuristics for treatment success, i.e., some reports associate treatment response with stronger rsFC, while others suggest the opposite. We suggest that the state of the pain connectome, rather than individual connections between its components, might serve as a more accurate functional determinant of treatment efficacy in chronic pain patient populations.

In the SCA, we found that more robust reduction in clinical pain intensity was predicted by (i) stronger rsFC of the aINS with the AG; (ii) weaker rsFC of the thalamus with the MFG; and (iii) weaker rsFC of the pgACC with the IPL. Curiously, we showed that rsFC in these regions also changed along with treatment response, and surprisingly a more pronounced reduction in BPI severity following M1-rTMS correlated with increases in rsFC of the thalamus with the MFG^21^. The thalamus plays an important role in pain processing both as a key brain area in the pain transmitting pathways (lateral nuclei) and in the modulation of pain (medial nuclei)^33^. The latter can be executed via the thalamic-prefrontal connection (specifically the mediodorsal nucleus^34^) and its level of connectivity is related to pain sensitivity^35,36^; intensity of induced pain in healthy subjects^37^; and with various chronic pain conditions such as migraine^38^, trigeminal neuropathic pain^39^, and postherpetic neuralgia^40^. In FMS, abnormal thalamus function^41^ and connectivity with prefrontal brain areas^16,42^ was identified during painful stimulation. Furthermore, regarding rsFC, increased thalamus-dorsal mPFC in FMS was associated with greater experimental pain sensitivity^43^. The mediodorsal nucleus serves as a primary relay offering major structural connections to the prefrontal cortex^44^ probably directly^45^ and indirectly (i.e., with connectivity via other thalamic nuclei) enabling extensive bilateral connections to the medio-frontal cortex^46^ via the anterior thalamic radiations^45^. Interestingly, a recent report revealed that the mediodorsal thalamic nuclei belong to the DMN^47^, thus further emphasizing the contribution of DMN connectivity to clinical pain intensity in FMS^5^. Taken together, our results suggest that reduced thalamo-prefrontal connectivity can predict rTMS positive effects on clinical pain by restoring its connectivity strength required to modulate pain.

Stronger connectivity between the aINS and the AG, another component of the DMN, also predicted rTMS-related analgesic effects. Living under chronic pain conditions is associated with functional alterations between the DMN components and between the DMN and the INS^5^, comprising the SN^4^. Yet, it is argued that it is not the presence of chronic pain that determines the patients’ DMN connectivity as compared to controls, but rather the current clinical pain intensity^5^. Specific to FMS, the strength of resting connectivity between the INS and DMN is positively correlated with clinical pain^48,49^ and decreased connectivity is associated with reductions in clinical pain following pregabalin administration^3^ and non-pharmacological intervention^48^. Thus, we add to the current knowledge about the role of the INS-DMN connectivity in FMS symptomology in that it also predicts treatment success, specifically M1-rTMS effects on clinical pain.

We found that weaker connectivity of the pgACC, a brain area that has a role in emotional regulation, with additional brain areas comprising the DMN i.e., the IPL, predicted the M1-rTMS treatment success in pain alleviation. The pgACC function is related to subjective emotional feelings and pain anticipation^50^. The pgACC is activated when individuals tend internally to their emotions^51^, and its activity is driven by interoceptive signals related to visceral and somatic pain^52^, thus assigning value to viscerosensory signals based on self-referential and conceptual knowledge^53^. In light of this, it is not surprising to see that the functional change of the pgACC is related to the pathophysiology of FMS^54^ and that pain-related negative affect is strongly associated with clinical pain intensity in FMS^55^. Anatomical connectivity patterns help to explicate the nature of its roles, as the pgACC shows strong functional connectivity with the DMN^53,56^, which is involved in self-referential processing and interoception, and its connectivity is associated with clinical pain intensity in FMS^9^.

Consistent with our hypothesis, we found that at the RSN level, weaker rsFC of the DMN-MFG and the ECN-rmPFC, predicted better reduction in clinical pain intensity. The DMN and ECN represent the neural substrates of opposite levels of attention. Connectivity within the DMN increases when an individual self-reflects and does not tend to an external situation, and the ECN is active when the requirement to act on an external factor arises, in parallel with DMN deactivation^57^. The right MFG, implicated in executive control, has been proposed to be a site of convergence of the two attentional networks, by serving as a circuit-breaker to interrupt ongoing endogenous attentional processes toward reorient attention to an exogenous stimulus^58^. Its role in pain is dependent on the source of pain. Namely, involvement in attention orientation away from experimentally induced painful stimuli in healthy individuals^59^ and in activation during hyperalgesia, whether experimentally induced^60^ or clinically manifested^61^. The rmPFC is explicitly involved in self-reflection^62^ and in appraisal of self-related information by assigning a positive or negative value to it^63^. It is a core hub of the DMN, and is activated during mind wandering and the resting state, both of which often involve self-referential processing^62^. Hence, the rmPFC’s role in emotion is related to the integration of self-referential and valence information^53^. Therefore, our results suggest that patients with DMN and ECN less occupied with areas that modulate attention towards pain, would benefit more from M1-rTMS. The RSN potential in predicting rTMS clinical effects has been previously reported in other clinical conditions such as depression^64^. This is coherent with the concept that rTMS may affect brain areas beyond the stimulated area through intra-network connections, as well as interactions between networks^65^.

We need to acknowledge some limitations on our data: (i) we excluded patients over the age of 55 years, as well as men suffering from FMS, and therefore cannot generalize our results to the entire FMS population; (ii) we used a flipped coil to introduce the sham condition, which, while being a prevalent approach in TMS studies, does not generate the typical somatosensory sensations of active stimulation, and might lead to bias in crossover study for individuals who already undergo the active TMS; and (iii) we focused on predicting the treatment effect following a 2-week intervention. Therefore, our prediction is restricted to the 2-week treatment period.

In summary, pre-rTMS resting-state connectivity between brain areas involved in pain processing (i.e., thalamus, aINS, pgACC) and components of the DMN (AG, IPL) and ECN (MFG) as well as between these networks and brain areas implicated in pain attentional modulation (MFG, mPFC) predicted better improvements in FMS-symptomatology. These results suggest that hub regions within DMN and ECN, both of which are functionally connected to M1 (the rTMS target)^66^ serve as candidate biomarkers for response prediction i.e., pain alleviation. Based on our recent report^21^ demonstrating that these clinical effects are linked to functional connectivity alteration in brain areas occupied with chronic pain, we suggest that the functioning of the RSN may constitute the connecting link between the stimulation areas i.e., M1 and the neurophysiological effects on distant pain brain areas that are involved in pain alleviation.

## Methods

This work is part of a more extensive neuroimaging-based study on the relationship between the structure and function of the brain and the clinical effects of M1-rTMS in FMS patients (ClinicalTrials.gov identifier—NCT02572726). This paper is based on a secondary analysis conducted on data presented previously^21^.

### Participants

All included patients were aged 19–55 years, met the 2010 American College of Rheumatology criteria for FMS diagnosis^67^, had been diagnosed with FMS by a qualified physician, and read and signed an informed consent form. We excluded individuals with: a familial history of epilepsy or pediatric febrile seizures; major depressive disorder; psychiatric conditions; attention-deficit hyperactivity disorder; metabolic disorders; obesity; cognitive impairment; claustrophobia; inability to provide informed consent, understand, or carry out the experiment’s instructions; pregnancy or lactation; or the presence of another pain condition other than FMS.

### Study design

This was a double-blinded, crossover, sham-controlled, and counter-balanced study. We investigated whether neuroimaging predicts the effect of M1-rTMS on clinical pain intensity, which was the primary clinical outcome in the research project. Participants were randomly allocated into one of 2 treatment groups: the first received real M1-rTMS treatment, followed by sham. We reversed the order in the other group. The participants and the study experimenter (Y.A.) were blinded to the treatment order. Before and immediately after each treatment series, participants underwent structural and resting-state neuroimaging scans and completed questionnaires regarding their clinical pain. Overall, each patient underwent 2 series of 10 daily treatments over 2 weeks. We maintained a washout period of at least 4 weeks between treatment series, based on evidence of the long-lasting effects of active rTMS on pain reduction up to 2 weeks following 10 daily sessions^18^. The institutional review board of the RAMBAM Healthcare Center reviewed and approved the study, in accordance with the decleration of Helsinki.

### Clinical pain questionnaires

As reported previously^21^, clinical pain was evaluated with the Hebrew versions of the Brief Pain Inventory (BPI)^68^ and McGill Pain Questionnaire (MPQ)^69^. From the BPI, we averaged the current and last 24 h’ pain intensity (*BPI-Severity*). From the MPQ, we summed up the 11 sensory descriptors (*MPQ-Sum Sensory)*, and current pain intensity rated on a 10-cm visual analog scale (*MPQ-VAS*).

### TMS intervention

Detailed descriptions of the preparation, experiment, and treatments appears in Ref.^21^, and in Supplementary Appendix A. Briefly, the real phase was consisted of 20 trains of ten 10 Hz stimulations at 80% of the pre-treatment resting motor threshold. The sham intervention had the same parameters, but with a flipped coil. Each session lasted 20 min.

### Image acquisition

We acquired high-resolution T1 structural images, and 300 resting-state fMRI volumes for each participant, before and after each treatment series. The full protocol is found in Supplementary Appendix B.

### Image preprocessing

#### Anatomical images

We used the CAT12 (http://www.neuro.uni-jena.de/vbm) and SPM12 toolboxes (Wellcome Trust Centre for Neuroimaging, London, UK; http://www.fil.ion.ucl.ac.uk/spm) for anatomical image preprocessing. Briefly, our preprocessing steps included: (i) segmentation into gray matter (GM), white matter (WM), and cerebrospinal fluid (CSF); (ii) co-registration to a mean study image using DARTEL; and (iii) normalization to a standard 1.5 × 1.5 × 1.5 mm voxel MNI152 space template.

#### Resting-state fMRI

The functional preprocessing pipeline described in Argaman et al.^21^. We used SPM8 for preprocessing the fMRI data (Wellcome Trust Centre for Neuroimaging, London, UK; http://www.fil.ion.ucl.ac.uk/spm). The preprocessing steps were: (i) manual rotation of images to a common plane; (ii) discarding the first 6 volumes to compensate for first scan signal fluctuations; (iii) slice-timing correction and realignment; (iv) extraction of the mean EPI image and a 6-direction motion parameter matrix for the scan, with none of the patients exceeding the ≥ 1.5 mm threshold in translation or 1.5° in rotation movement in each direction; (v) coregistration of functional volumes to the structural image; (vi) normalization to a standard MNI152 EPI template with 3 × 3 × 3 mm voxels; and (vii) smoothing with a 6 mm—full width at a half-maximum (FWHM) Gaussian kernel. Next, in CONN 18b^70^, we created patient-specific binary and eroded GM masks, applied a 0.01–0.1 Hz bandpass filter, and removed nuisance effects of WM, CSF, and motion artifacts with linear regression with Component-Based Noise Correction. Finally, we removed signal drift, sudden signal fluctuations, and voxel-wise correlations using linear detrending and despiking. We used the resulting voxel-specific timecourses for subsequent group independent component analysis (gICA) and seed-based correlation analysis (SCA).

### rsFC analyses

#### gICA

gICA was performed as previously described^71^. Briefly, voxel-wise data from all participants were concatenated to create group maps. Then, principal component analysis (PCA) was used to reduce data into 64 principal components. Then, group data reduction was performed using the Fast-ICA algorithm, resulting in 25 independent components (IC). Finally, in the back-reconstruction step, timecourses from each IC were regressed onto the individual participants’ spatial maps, yielding individual beta maps for each IC and each participant. The rsFC of each network was calculated for each individual, based on the connectivity of each voxel within the map to every other voxel in the brain. The DMN, SN, and ECN were identified by trained individuals (I.W.F. and Y.A.) and by calculating the spatial match between our ICs and a priori RSN templates using Dice’s Similarity Coefficient.

#### SCA

We placed 6 mm radius spheres (except the amygdala, for which we used 3 mm spheres) in regions grounded on their role in ascending nociceptive processing or descending pain inhibition, based on previous evidence of their pain-related connectivity or activity as described in detail in Argaman et al.^21^. For the descending antinociceptive system we defined specific coordinates for seeds in the following (x, y, z) coordinates in MNI space: the pgACC (± 7, 39, − 2)^50^, the amygdala (± 22, 2, − 20)^72^, the aINS (± 35, 16, 3)^73^, the dlPFC (± 40, 34, 40) within Brodmann area 46^74^, and the ventro-medial PFC (vmPFC) (± 9, 56, − 12)^75^. For ascending nociceptive system assessment, we placed seeds in the S1 (± 30, − 37, 68)^50^, entire thalamus (atlas)^76^, and pINS (± 38, − 11, 7)^73^. For the pgACC and vmPFC, we used the average timecourses of both hemispheres due to the seeds’ proximity to the midline. We investigated the connectivity of each ROI with the entire brain since FMS is considered a chronic pain disorder, and its pathophysiology and symptomatology are anchored in widespread functional connectivity changes with brain areas outside the ascending and modulatory pain pathways^8,49,77,78^.

We produced condition- and subject-specific first-level statistical T-maps. In gICA, these maps represent the individual independent components of each DMN, SN, ECN, or the probability that each voxel belongs to a specific network; while in SCA, they represent the correlation coefficients of each pre-selected seed’s timecourse with those of every other voxel in the brain.

### Statistical analysis

We used JMP 14 (SAS, Cary, NC, USA) and R 3.5.1 running on an RStudio 1.0.153 platform (R Core Team 2016. R: A language and environment for statistical computing, R Foundation for Statistical Computing, Vienna, Austria) for our statistical analyses. Briefly, we tested the clinical pain outcome measures for time × treatment interactions using a repeated-measures ANOVA model or Friedman’s Test. Our post-hoc tests consisted of paired t-tests or Wilcoxon–Nemenyi–McDonald–Thompson paired tests for nonparametric models. We multiplied all post-hoc p-values by 4 to accommodate the post-hoc tests based on the number of pre-planned 2-tailed contrasts^21^. Thus, we focused on outcome measures that had a significant time × treatment interaction that significantly differed between post-real vs. pre-real but not post-sham vs. pre-sham contrasts or post-real vs. post-sham contrast.

We aimed to determine whether significant changes in clinical pain, namely, MPQ-Sensory, MPQ-VAS, and BPI-Severity following real but not sham M1-rTMS (as described in the previous paragraph) could be predicted by pre-treatment brain rsFC. We therefore used separate general linear models for group-level analyses, which we set up for each clinical pain measure that significantly changed following real and not sham M1-rTMS. The dependent variable was a vector of individual treatment responses (post-minus-pre-treatment values), while the independent variable was a vector of the first-level pre-real treatment statistical T-maps. In all analyses, we used a voxel-wise threshold of p < 0.001 and a cluster-level false-discovery rate (FDR) corrected threshold of p < 0.05. In SCA, we utilized parametric tests, and in gICA, we used nonparametric statistics using permutation tests, shuffling the labels of the tested data randomly 10,000 times and generating a maxT distribution. Also, in SCA, we multiplied the significance values of each resulting cluster by the number of ROIs (6 bottom-up, and 8 top-down). We report R-to-Z Fisher-transformed seed/network-to-cluster correlation values from gICA and SCA, which were averaged across all the voxels of the significant clusters. Finally, we examined whether rsFC predicted treatment response of 1 treatment type but not the other. For this, we obtained the cluster rsFC R-to-Z-transformed values from clusters exhibiting significant association between pre-treatment network/seed connectivity changes in clinical pain scores following real or sham M1-rTMS, similar to the process described in Ref.^21^. Finally, to find how pre-treatment connectivity relates to the clinical effect in each treatment type, we plotted the clinical effect (post-minus-pre-treatment values) against the pre-treatment rsFC values, for each treatment, and calculated the correlation values for each treatment type separately.

Finally, we visualized the final significant target connectivity clusters in both analyses by rendering them unto a standard MNI152 template using MRIcroGL (Chris Rorden, https://www.nitrc.org/projects/mricrogl/).

## Supplementary Information

Supplementary Information.

## Data Availability

Data are available upon request from I.W.F.
