# Supplementary material for: Resting-state functional connectivity predicts motor cortex stimulation-dependent pain relief in fibromyalgia syndrome patients
Source: Sci Rep. 2022 Oct 12;12:17135. doi: 10.1038/s41598-022-21557-x (PMC9556524; doi:10.1038/s41598-022-21557-x)
Supplement: Supplementary file 1 — Supplementary Information. [file 41598_2022_21557_MOESM1_ESM.pdf]

Resting-State Functional Connectivity Predicts Motor Cortex Stimulation-Dependent Pain Relief in Fibromyalgia Syndrome Patients

Yuval Argaman, Yelena Granovsky, Elliot Sprecher, Alon Sinai, David Yarnitsky, Irit Weissman-Fogel

Supplementary Information

Appendix A – M1-rTMS Intervention

Patients sat upright comfortably, we placed a lycra cap on their scalps, and provided them with earplugs for the experiment and treatment duration. We marked the approximate location of the right M1 on the cap. Then we thoroughly cleaned the left palm and volar forearm's skin and attached 2 electrodes to the left hand in a belly-tendon configuration, over the left Adductor Policis Brevis (APB) muscle, with a grounding electrode on the volar forearm. Next, we probed the region around the right M1 with single pulses from an 8-figure coil (MCF-B65, MagVenture, Farum, Denmark) at 60% of the maximal amplitude of the device (MagPro X-100, MagVenture, Farum Denmark). We repeated this process until a pulse elicited a discernable muscle twitch of the left APB. If no noticeable ABP twitch had been elicited, we gradually increased the stimulation intensity. Patients who had no APB response at an intensity of >80% maximal device amplitude were subsequently excluded from the study. Then, to find the resting motor threshold (rMT), we lowered the pulse amplitude so that it elicited no ABP twitch. We gradually increased the pulse amplitude until producing a muscle-evoked potential with an amplitude of at least 50 microvolts in at least 5/10 attempts, defined as the rMT. The cutoff stimulation amplitude was 65%, which corresponds to 80% of 80%.

Each treatment included 2000 pulses at 80% rMT in 20 trains of one-hundred 10-Hz pulses and 50-sec intervals between trains. We performed sham stimulation by flipping the coil upside down so its passive cooling unit faced the skull and the magnets were away from it 39,40. The treatments were conveyed by trained laboratory members who were not blinded to the experimental conditions, delivered

the treatments, and were present in the room with the patients to monitor side effects.

Appendix B – Image Acquisition Protocol

During the imaging session we provided participants with earplugs to minimize scanner noise. We fitted participants with foam pads to minimize head motion. In T1-weighted structural images, we utilized a spoiled gradient recall sequence with 1×1×1 mm voxels, a flip angle of 120, a matrix size of 256×256, 172 slices, a field of view (FOV) of 25.6 cm, and a receiver bandwidth of 83.33 Hz. Next, 300 resting-state fMRI volumes were acquired for 10 min, using a whole-brain gradient echo-planar imaging (EPI) sequence, 3.4×3.4×3.4 mm voxels, TR = 2000 ms, TE = 30 ms, flip angle = 750, matrix size = 64×64 mm, slices = 43, FOV of 22 cm, and a receiver bandwidth of 31.25 Hz. During resting-state fMRI, we requested patients to stay awake with eyes open to the best of their ability and think of nothing in particular.
